# Measuring extracellular volume fraction by MRI: First verification of values given by clinical sequences

**DOI:** 10.1002/mrm.27938

**Published:** 2019-08-16

**Authors:** David Nordlund, Christos Xanthis, Sebastian Bidhult, Robert Jablonowski, Mikael Kanski, Sascha Kopic, Marcus Carlsson, Henrik Engblom, Anthony H. Aletras, Håkan Arheden

**Affiliations:** ^1^ Department of Clinical Physiology, Clinical Sciences Lund University and Lund University Hospital Lund Sweden; ^2^ Laboratory of Computing and Medical Informatics School of Medicine Aristotle University of Thessaloniki Thessaloniki Greece; ^3^ Department of Biomedical Engineering Faculty of Engineering Lund University Lund Sweden

**Keywords:** ECV, extracellular volume, MOLLI, SASHA, T1‐mapping

## Abstract

**Purpose:**

To verify MR measurements of myocardial extracellular volume fraction (ECV) based on clinically applicable T1‐mapping sequences against ECV measurements by radioisotope tracer in pigs and to relate the results to those obtained in volunteers.

**Methods:**

Between May 2016 and March 2017, 8 volunteers (25 ± 4 years, 3 female) and 8 pigs (4 female) underwent ECV assessment with SASHA, MOLLI5(3b)3, MOLLI5(3s)3, and MOLLI5s(3s)3s. Myocardial ECV was measured independently in pigs using a radioisotope tracer method.

**Results:**

In pigs, ECV in normal myocardium was not different between radioisotope (average ± standard deviation; 19 ± 2%) and SASHA (21 ± 2%; *P* = 0.086). ECV was higher by MOLLI5(3b)3 (26 ± 2%), MOLLI5(3s)3 (25 ± 2%), and MOLLI5s(3s)3s (25 ± 2%) compared with SASHA or radioisotope (*P* ≤ 0.001 for all). ECV in volunteers was higher by MOLLI5(3b)3 (26 ± 3%) and MOLLI5(3s)3 (26 ± 3%) than by SASHA (22 ± 3%; *P* = 0.022 and *P* = 0.033). No difference was found between MOLLI5s(3s)3s (25 ± 3%) and SASHA (*P* = 0.225). Native T1 of blood and myocardium as well as postcontrast T1 of myocardium was consistently lower using MOLLI compared with SASHA. ECV increased over time as measured by MOLLI5(3b)3 and MOLLI5(3s)3 for pigs (0.08% and 0.07%/min; *P* = 0.004 and *P* = 0.013) and by MOLLI5s(3s)3s for volunteers (0.07%/min; *P* = 0.032) but did not increase as measured by SASHA.

**Conclusion:**

Clinically available MOLLI and SASHA techniques can be used to accurately estimate ECV in normal myocardium where MOLLI‐sequences show minor overestimation driven by underestimation of postcontrast T1 when compared with SASHA. The timing of imaging after contrast administration affected the measurement of ECV using some variants of the MOLLI sequence.

## 1 INTRODUCTION

Tissue such as myocardium maintains a clear distinction between intracellular and extracellular fluid compartments. Extracellular volume fraction (ECV) is affected by disease processes, including diffuse myocardial fibrosis, general edema from myocarditis, or myocardial storage disease, and could potentially be used to identify and grade the severity of these pathologies. Measurements of ECV have historically only been possible using experimental techniques,^1^, ^2^, ^3^ precluding any clinical applicability.

Cardiovascular MR (CMR) can be used to accurately measure ECV by comparing T1 recovery‐times measured before and after contrast administration^4^, ^5^ based on the assumptions that (1) the T1 relaxation‐times change in proportion to the concentration of contrast agent present, and (2) the contrast agent distributes in proportion to the extracellular space. These early techniques relied on slow MR protocols not suitable for clinical use. More recently, technical advancements have allowed for faster sequences for measuring T1 relaxation‐times, which can be used clinically and show potential for measuring ECV.^6^, ^7^, ^8^ Commonly used faster T1‐mapping techniques include MOdified Look‐Locker Inversion recovery (MOLLI)^6^ and SAturation recovery single SHot Acquisition (SASHA).^9^

The major advantage of measuring ECV is that it supplies an absolute, physiological value as opposed to conventional CMR methods which rely on relative differences in signal intensity within the myocardium. This means that ECV measurements could improve the ability to detect diffuse processes affecting the entire myocardium and subtle changes which may not be readily apparent by visual assessment. The usefulness of this method, specifically to differentiate disease from health in unclear cases, is therefore highly dependent upon accurate and precise measurements of normal values, whereas this is of less importance in clear cases of disease. While it is currently known that different T1‐mapping sequences produce different ECV values,^10^ it is not known which specific sequence yields the most accurate results. Therefore, there is a need for verification of ECV measurement techniques using an independent reference standard.

The first experimental studies^4^, ^5^ verified the accuracy and precision of MR derived ECV of normal and infarcted myocardium^4^, ^5^ and in myocardium at risk^5^ by measuring the activity of injected radioactive isotope (^99m^Tc), bound to an extracellular carrier molecule (DTPA). Activity measurements were normalized to the activity of plasma, serving as a 100% ECV calibration point to calculate ECV independently of CMR measurements. Clinical sequences, however, have not been verified against an independent reference standard to date.

The aim of this study was, therefore, to verify ECV measurements obtained with clinically applicable T1‐mapping techniques, including SASHA and variants of MOLLI, in a porcine model against a radioisotope‐based reference standard and to relate the results to those obtained by the same pulse sequences in healthy human volunteers.

## 2 METHODS

The data in this study were collected between May 2016 and March 2017. The experimental part of the study was approved by the regional ethics committee for animal experiments (registration number M94‐14), whereas the volunteer part was approved by the regional ethics committee in Lund, Sweden, and written informed consent was acquired from all subjects (registration number 2013/891).

### 2.1 Experimental protocol

Pigs of 40‐50 kg (see characteristics in Table 1) were sedated using ketamine 15 mg/kg (Ketaminol, Intervet, Danderyd, Sweden) and midazolam 0.5 mg/kg (Dormicum, Roche AB, Stockholm, Sweden) administered intramuscularly. Propofol (20 mg/mL) (Propofol Sandoz AS, Copenhagen, Denmark) was administered and intubation was performed using a cuffed endotracheal tube after which mechanical ventilation, regulated to a pCO2 of 5.0‐6.0 kPa, was established. Inhalation of Sevoflurane gas (Sevorane, Baxter Medical AB, Kista, Sweden) was used to maintain anesthesia by means of a disposable administration system (AnaConDa, Sedana Medical AB, Uppsala, Sweden).

**Table 1:** Pig and healthy volunteer characteristics

	Pigs	Volunteers
Height, cm	–	178 ± 9
Weight, kg	46 ± 2	69 ± 8
Age, years	–	25 ± 4
Female, %	50	38
Heart rate, beats/min	88 ± 11	64 ± 11
Hct, %	31 ± 4	40 ± 4

Venous and arterial access was established by inserting 6Fr introducer sheaths in the femoral artery and vein after which 10,000 IU of heparin (LEO Pharma AB, Malmö, Sweden) was administered. Animals were monitored with regards to arterial blood pressure, heart rate, electrocardiogram, pulse‐oximetry, temperature, and arterial blood gas. During the experiments a slow infusion of Ringer's Acetate (Fresenius Kabi AB, Uppsala, Sweden) was maintained. Nondepolarizing muscle relaxant (Rocuronium, B. Braun Medical AB, Danderyd, Sweden) was administered as needed to minimize respiratory artefacts during imaging.

After sedation and preparation, the pigs were transported to the MR department for imaging. Blood was sampled for hematocrit (Hct) at arrival, before, 30 min after, and 60 min after contrast agent‐injection to ensure Hct did not change between pre‐ and postcontrast T1‐map acquisitions.^11^ Hct samples were analyzed at an accredited laboratory (ISO/IEC 17025:2005). After MR imaging, 1000 MBq of ^99m^Tc‐DTPA was administered intravenously and allowed to circulate for 15 min. The animals were then euthanized by infusion of a saturated potassium chloride (KCl) solution, after which hearts were explanted and sliced into 5‐ to 10‐mm‐thick slices. One 3‐mm‐diameter punch biopsy was taken from each of 16 sectors in 1 basal, 1 mid‐ventricular, and 1 apical slice according to the AHA 17‐segment model,^12^ excluding the most apical sector.

### 2.2 Healthy volunteers

Volunteers with no history of systemic or cardiac disease were included (see characteristics in Table 1). Blood for Hct analysis was sampled from volunteers after 1 h of lying down, just before contrast administration.

### 2.3 MR imaging

MR imaging was carried out on a Magnetom Aera 1.5T scanner (Siemens Healthcare, Erlangen, Germany) using an 18‐channel receiver coil optimized for CMR applications (body array and spine array). Three MOLLI^6^, ^13^, ^14^, ^15^ protocols and 1 SASHA^9^ protocol based on a prototype sequence (see Table 2 for details) were used to acquire images before injection of 0.2 mmol/kg gadolinium (Gd)‐DOTA (Dotarem, Guerbet, Roissy, France) and repeatedly over 1 h postcontrast. One mid‐ventricular short‐axis slice was acquired for all protocols and at all time‐points. For pigs, retrospectively gated balanced steady‐state free precession (bSSFP) images and late Gd enhancement images were acquired after contrast injection, including short‐axis stacks covering the entire left ventricle as previously described.^16^, ^17^ For volunteers, a full short‐axis stack of bSSFP images was acquired after contrast injection. All images were acquired during end‐expiratory suspended respiration.

**Table 2:** T1‐mapping sequences used^a^

Sequence parameters	MOLLI 5(3b)3	MOLLI 5(3s)3	MOLLI 5s(3s)3s	SASHA
Precontrast scheme	5(3b)3	5(3s)3	5s(3s)3s	10xSAT images + 1xREF image
Postcontrast scheme	4(1b)3(1b)2	4(1s)3(1s)2	4s(1s)3s(1s)2s	10xSAT images + 1xREF image
Flip angle [°]	35	35	35	70
TE/TR [ms]	1.1/2.6	1.1/2.6	1.1/2.6	1.1/2.6
Pixel size [mm^2^]	1.9 × 1.9	1.9 × 1.9	1.9 × 1.9	1.9 × 1.9
Slice thickness [mm]	6	6	6	6
Field of view [mm^2^]	360 × 270	360 × 270	360 × 270	360 × 270
Parallel imaging factor (GRAPPA)	2	2	2	2
Number of phase encoding steps	63	63	63	63
Readout duration [ms]	161	161	161	161
Readout type	bSSFP	bSSFP	bSSFP	bSSFP
Receiver BW [kHz]	208.32	208.32	208.32	208.32
Typical breathhold duration for animals [s]	7.5 (Pre & Post)	8.4 (Pre)	11 (Pre & Post)	7.5 (Pre & Post)
		8.1 (Post)		
Typical breathhold duration for volunteers [s]	10 (Pre & Post)	10.5 (Pre)	11 (Pre & Post)	10 (Pre & Post)
		10.4 (Post)		

Abbreviations: BW, bandwidth; GRAPPA, generalized autocalibrating partial parallel acquisition; TE/TR, echo time/repetition time.

a Sequence parameters of MOLLI^14^, ^15^, ^35^ and SASHA^9^ schemes used pre‐ and postcontrast, respectively. Numbers not in parentheses are number of acquisitions after the inversion or, if followed by “s” it is the number of seconds during which acquisition is happening. Numbers in parentheses show the length of the pause before the next inversion where “b” denotes the pause as the number of beats while “s” denotes the pause as the number of seconds.

### 2.4 Radioisotope measurements

Radioactive counts were measured from the punch biopsies taken from the explanted porcine hearts and from plasma as well as whole blood after weighing each sample separately. Visible droplets of blood were gently removed from the biopsies taking care to avoid the effect of capillary action on the myocardium. An automatic gamma counter (WALLAC Wizard 3, PerkinElmer, Upplands Väsby, Sweden) was used to measure radioactivity over 5 min, adjusting measures for the half‐time of 99mTc. Samples were corrected for detector dead time and rerun if dead time exceeded 10%. Weighing was performed using calibrated precision scales (AB104, Mettler Toledo, Stockholm, Sweden) with a least measurable weight of 0.097 g at an allowed error of 0.1%. The number of counts in the myocardial biopsies was normalized to their respective weight. The average value of all 16 biopsies were used for each pig. The activity of plasma was used as a reference of 100% ECV. ECV fraction was calculated by dividing the weight‐normalized activity (counts) of the myocardial biopsies with the weight‐normalized activity of plasma:

(1) $$ECVf=\frac{{\text{counts}}_{\text{biopsy}}/{\text{weight}}_{\text{biopsy}}}{{\text{counts}}_{\text{plasma}}/{\text{weight}}_{\text{plasma}}}.$$

The activity of whole blood was normalized to the activity of plasma to calculate Hct and was compared with directly measured Hct values as an internal quality control. For 1 pig, there was visible hemolysis in the plasma sample. For this pig, the radioactivity in whole blood was divided by 1 minus Hct as given by the laboratory to be used as the plasma reference. ECV fraction for this pig was 21%.

An additional experiment was performed to determine whether the time from termination and explantation of the heart was of importance.

Immediately after termination with KCl, a chunk of the left ventricular anterolateral wall was excised, from which a sample was put in a test tube and weighed. This procedure was repeated 17 times over the first 10 min and an additional 5 times over the first hour. The radioactivity of the samples was then measured as described above and later normalized to the weights of the samples.

### 2.5 Image analysis

CMR images were analyzed using Segment 2.0 R5453 (Medviso, Lund, Sweden).^18^ T1‐measurements were performed in the following steps: after applying motion correction, endo‐ and epicardium were manually delineated for 1 inversion time (TI) in 1 acquisition for each subject and the delineation was then copied to all acquisitions and for images of all TIs/saturation times. A mid‐septal region of interest (ROI) was defined as 60° of the left ventricular circumference using the middle 60% of the wall thickness. Septal ROIs were chosen as there are theoretical arguments that the higher through‐plane motion in the lateral wall might affect T1 measurements.^19^ Mean intensities within the ROIs were then plotted against TI and T1 was calculated using a 3‐parameter nonlinear fit. The Look‐Locker correction was applied for all T1‐estimates of myocardium extracted from MOLLI pulse sequences.^20^ T1‐values were measured before and after contrast injection and ECV was calculated using Equation 2 as initially described^4^:

(2) $$\left(1-Hct\right)\times \frac{1/\text{Myocardial T}1_{\text{post contrast}}-1/\text{Myocardial T}1_{\text{pre contrast}}}{1/\text{Blood T}1_{\text{post contrast}}-1/\text{Blood T}1_{\text{pre contrast}}}.$$

Late Gd enhancement and contrast‐enhanced bSSFP images were visually assessed to ensure no focal myocardial edema or fibrosis was present in the pigs.

### 2.6 Statistical analysis

Statistical analyses were performed using SPSS Statistics Viewer (IBM Corp., Armonk, NY). Linear regression was performed to analyze changes in ECV over time after contrast, both including and excluding data from the first 10 min after contrast injection to account for the time it takes to equilibrate Gd over different compartments.^21^, ^22^ Linear regression was also used to analyze changes in ECV by radioisotope over time after termination. Repeated‐measures analysis of variance followed by Bonferroni multiple comparisons test was used to determine whether there were statistically significant differences between ECV by radioisotope and ECV by T1‐measurements, using values from 10 to 20 min after contrast injection, and whether there were differences in Hct at baseline, 30 min after, and 60 min after contrast injection. One‐way analysis of variance was also used to test for differences in ECV by radioisotope between slice positions. Modified Bland‐Altman analysis was used to analyze differences between measurement methods, using ECV by radioisotope as reference standard.^23^ A *P*‐value < 0.05 was considered to indicate statistical significance. Results are presented as mean ± standard deviation. T1‐values are presented to the nearest 10 ms to improve readability, reflecting that there is currently no reasonable way of achieving higher precision than that.

## 3 RESULTS

### 3.1 Population

Eight pigs and 8 volunteers were included; see Table 1 for characteristics.

### 3.2 ECV by CMR and radioisotope

**Figure 1:**
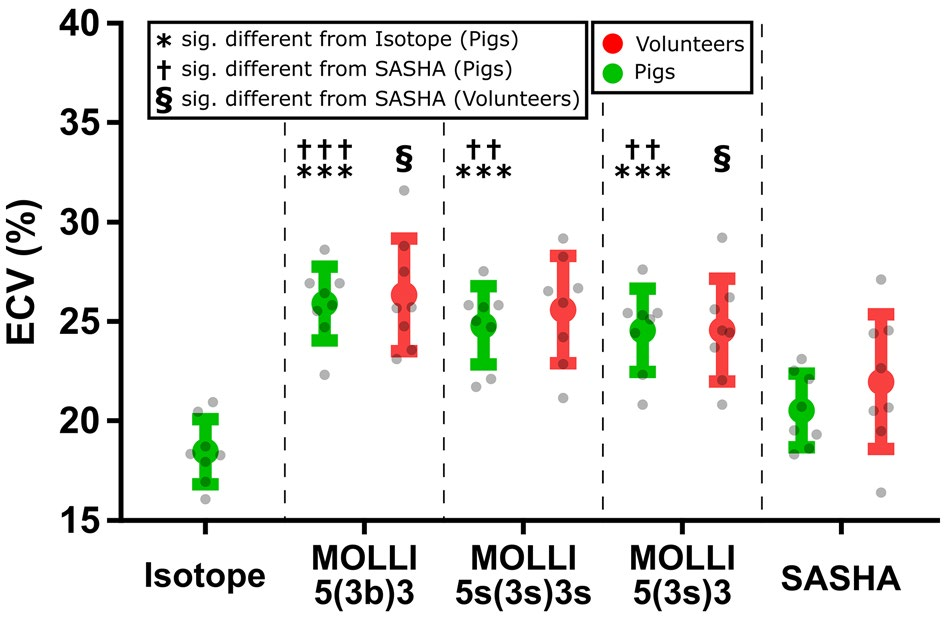
ECV in pigs by CMR and isotope and in volunteers by CMR. Note the higher ECV as measured by MOLLI versus both isotope and SASHA in pigs and versus SASHA in volunteers. The error bars show mean ± standard deviation. One symbol: *P* < 0.05. Two symbols: *P* < 0.01. Three symbols: *P* < 0.001

**Figure 2:**
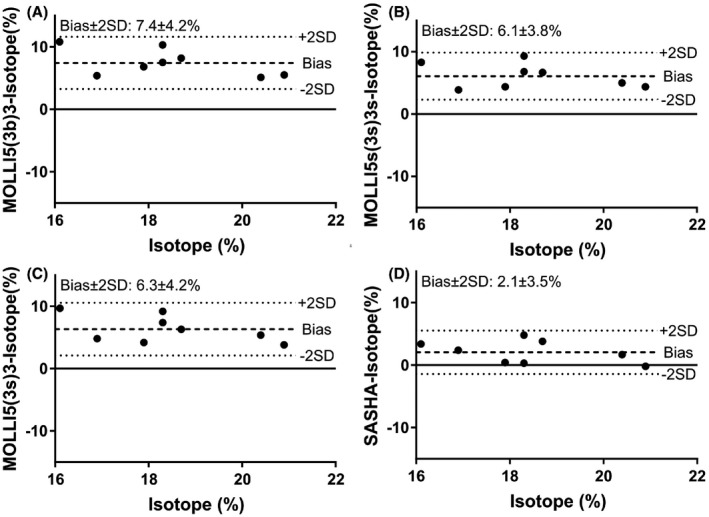
Modified Bland‐Altman plots of the difference in ECV by radioisotope and different T1 mapping techniques on the y‐axis and ECV by radioisotope on the x‐axis using data from 8 pigs. Note that SASHA shows a lesser bias than MOLLI compared with the reference method

ECV results by radioisotope and CMR are shown in Figure 1 and bias is shown in Figure 2. The mean weight of biopsies was 0.24 ± 0.11 g. Mean weight of plasma and whole blood was 0.24 ± 0.05 g. ECV in healthy myocardium of pigs was found to be 18 ± 2% by radioisotope, 26 ± 2% by MOLLI 5(3b)3, 25 ± 2% by MOLLI 5(3s)3, 25 ± 2% by MOLLI 5s(3s)3s, and 21 ± 2% by SASHA. No statistically significant difference was seen between SASHA and radioisotope (mean difference 2%; *P* = 0.150) while MOLLI 5(3b)3, MOLLI 5(3s)3, and MOLLI 5s(3s)3s all showed significantly higher ECV than both radioisotope (mean difference: 7%, 6%, and 6%; *P* < 0.001, *P* = 0.001, *P* < 0.001) and SASHA (mean difference: 5%, 4%, 4%; *P* < 0.001, *P* = 0.002, *P* = 0.001).

Similar to this, ECV in the myocardium of healthy human volunteers was 26 ± 3% by MOLLI 5(3b)3, 26 ± 3% by MOLLI 5(3s)3, 25 ± 3 by MOLLI 5s(3s)3s and 22 ± 3% for SASHA. There was a statistically significant difference of 4% between MOLLI 5(3b)3 and SASHA as well as between MOLLI 5(3s)3 and SASHA (*P* = 0.022, *P* = 0.033) while MOLLI 5s(3s)3s showed no significant difference from SASHA (mean difference: 3%, *P* = 0.225). No difference in ECV by radioisotope was seen between slice positions (apical: 19 ± 2%; mid: 18 ± 2%; basal: 18 ± 2%; *P* = 0.363).

### 3.3 T1‐values

**Figure 3:**
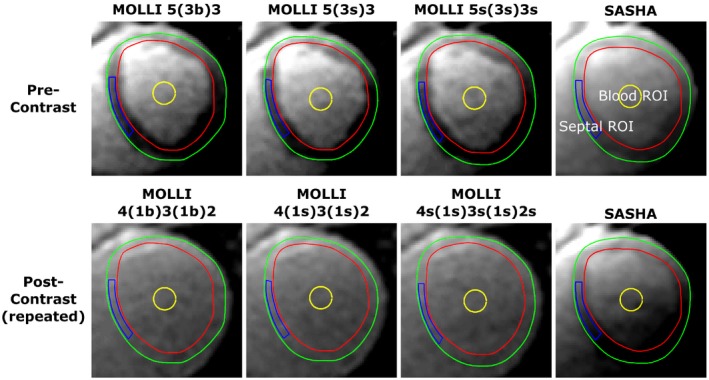
Example images of the first TI in the series for pre‐ and postcontrast T1‐mapping sequences. The postcontrast images are taken from 0 to 10 min after contrast administration. Regions of interest were added for blood (yellow) and myocardial septum (blue) and then copied to all TIs for all acquisition time points in that subject. Red lines show endocardium and green lines show epicardium

**Figure 4:**
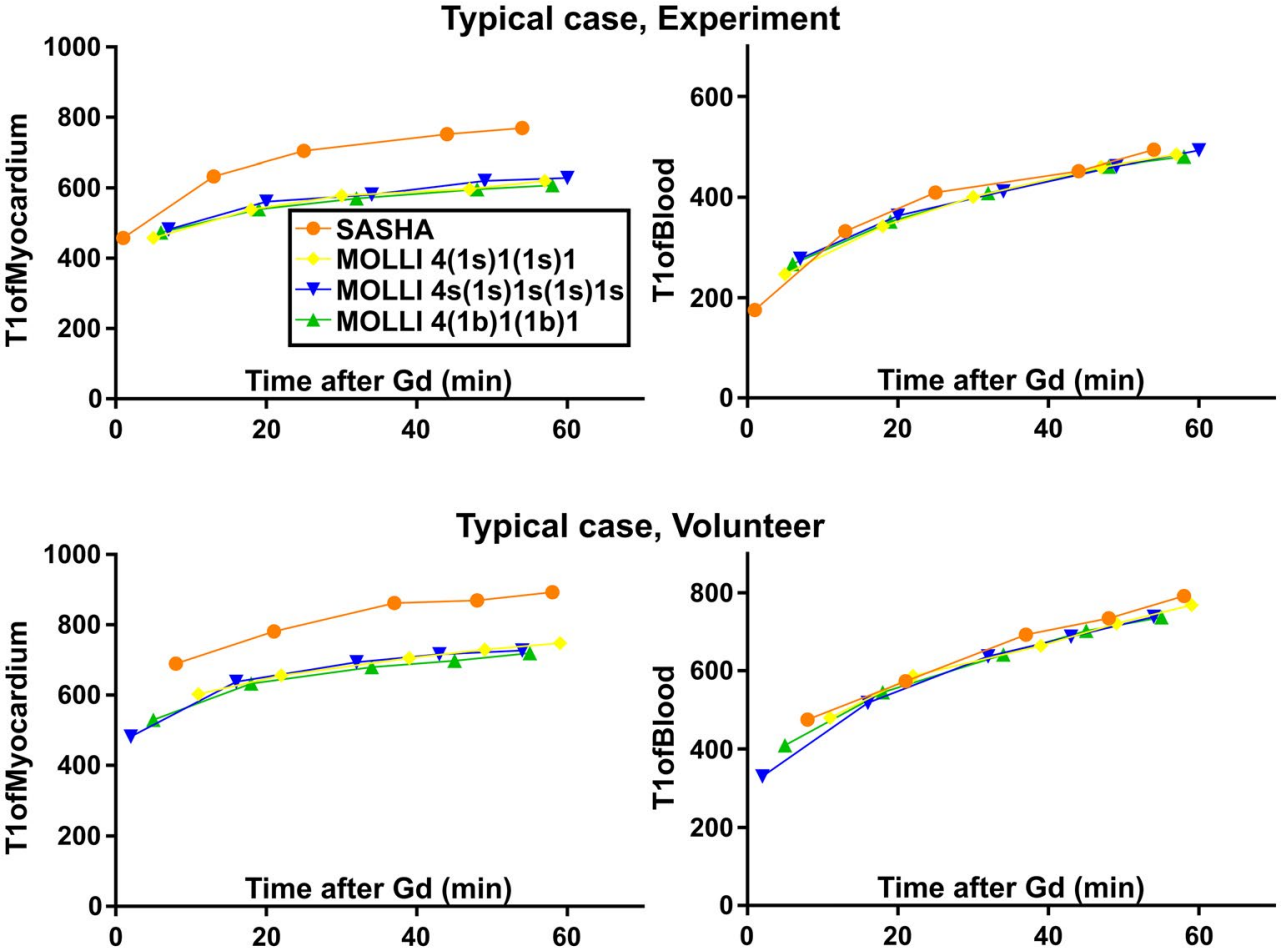
Typical case in a porcine experiment and in a volunteer of postcontrast T1 values for myocardium to the left and blood to the right. Note that the MOLLI sequences show lower T1 in myocardium compared with SASHA and that there is no difference in T1 between the sequences for blood. For data on all the experiments and volunteers, see Supporting Information Figures S7 and S8

Example images from the T1‐mapping sequences are shown in Figure 3. Native T1‐times in pigs and volunteers are seen in Table 3. For postcontrast T1 values of blood and of myocardium, please see Figure 4 for typical cases and Supporting Information Figures S7 and S8, which are available online, for the complete set of data. Native T1‐times of blood in pigs were significantly higher when measured by SASHA compared with MOLLI 5(3b)3, MOLLI 5(3s)3, and MOLLI 5s(3s)3s (mean difference: 220 ms, 200 ms, 160 ms; *P* = 0.001, *P* = 0.002, *P* = 0.010, respectively). Native T1‐times of myocardium in pigs were also significantly higher by SASHA than for the MOLLI sequences (mean differences: 210 ms, 190 ms, 190 ms; *P* < 0.001 for all). A similar pattern was seen in volunteers where SASHA showed statistically higher T1‐times of blood compared with MOLLI sequences (mean differences: 120 ms, 120 ms, 120 ms; *P* = 0.020, *P* = 0.005, *P* = 0.003, respectively) as well as higher T1‐times of myocardium (mean differences: 240 ms, 240 ms, 240 ms; *P* < 0.001 for all).

**Table 3:** Native T1‐values

	MOLLI 5(3b)3	MOLLI 5(3s)3	MOLLI 5s(3s)3s	SASHA
Pigs				
Native T1, blood	1600 ± 60 ms	1620 ± 60 ms	1660 ± 70 ms	1820 ± 110 ms
Native T1, myocardium	960 ± 40 ms	980 ± 50 ms	980 ± 40 ms	1170 ± 40 ms
Volunteers				
Native T1, blood	1510 ± 70 ms	1510 ± 80 ms	1510 ± 80 ms	1630 ± 90 ms
Native T1, myocardium	980 ± 20 ms	980 ± 10 ms	980 ± 20 ms	1220 ± 30 ms

### 3.4 Timing of imaging after contrast injection

**Figure 5:**
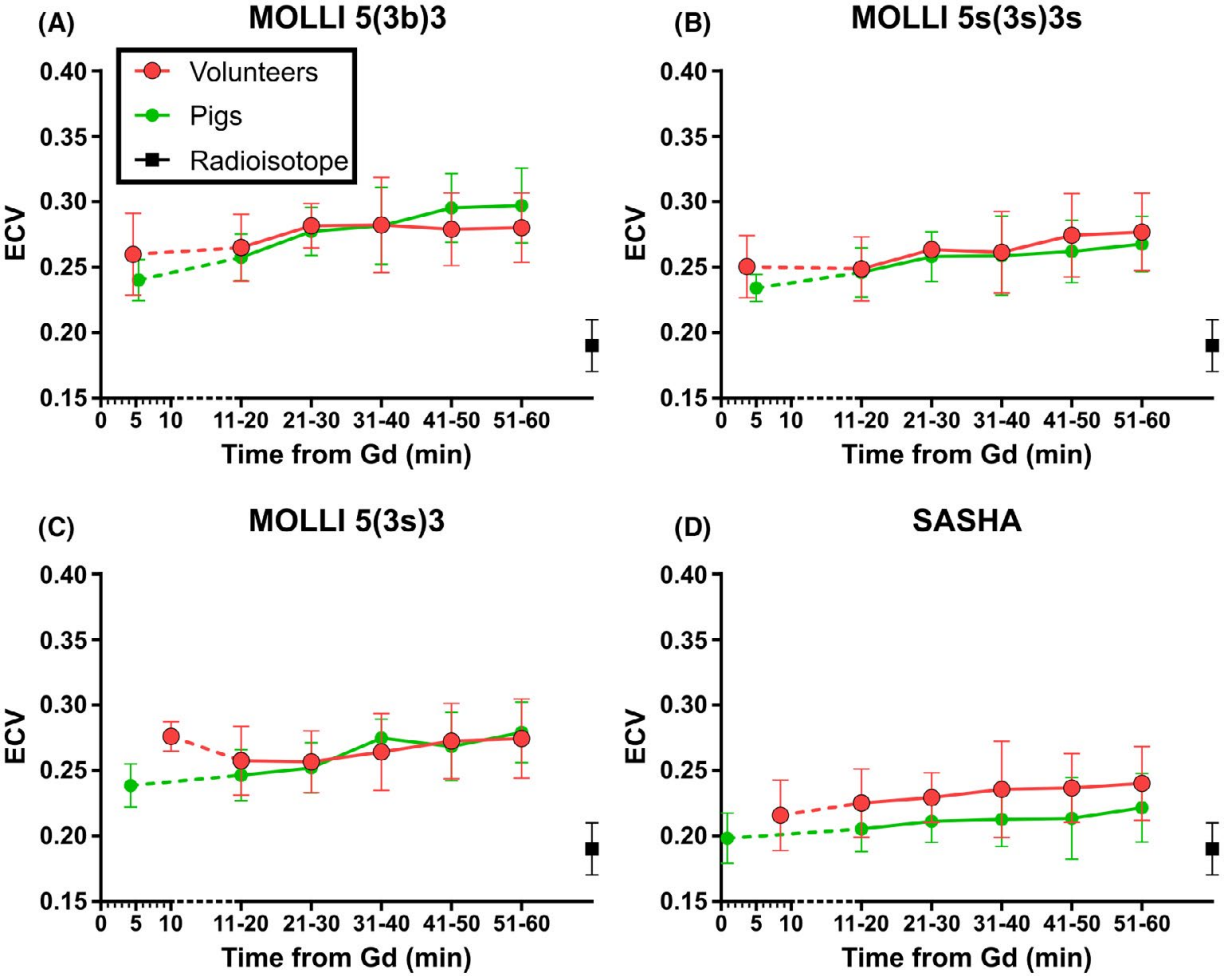
ECV over time after Gd calculated using 4 different T1‐mapping sequences. Red shows ECV of healthy volunteers (*n* = 8), green shows ECV of pigs (*n* = 8), and the black box shows ECV as measured by radioisotope in pigs. The first ECV‐measurements are shown at the actual mean time of acquisition while, for visualization purposes, the following data points are shown at intervals of 10 min. Error bars show mean ± standard deviation. Note that all MOLLI sequences (panels A‐C) show consistently higher ECV compared with the radioisotope reference method while SASHA agrees more closely with radioisotope. For information on individual data of ECV by sequence for each experiment and volunteer, please see Supporting Information Figure S6

The ECV plotted against time after contrast injection for pigs and volunteers is shown in Figure 5 and Supporting Information Figure S6. For pigs, there was an increase in ECV over time after contrast injection for MOLLI 5(3b)3 (0.11%/min; y‐intercept = 0.24; r^2^ = 0.42; *P* < 0.001), MOLLI 5(3s)3 (0.07%/min; y‐intercept = 0.22; r^2^ = 0.29; *P* < 0.001), MOLLI 5s(3s)3s (0.05%/min; y‐intercept = 0.22; r^2^ = 0.19; *P* = 0.050), and SASHA (0.04%/min; y‐intercept = 0.20; r^2^ = 0.11; *P* = 0.036). When excluding data from the first 10 min from the analysis, there was still an increase in ECV over time for pigs using MOLLI 5(3b)3 (0.08%/min; y‐intercept = 0.25; r^2^ = 0.22; *P* = 0.004) and MOLLI 5(3s)3 (0.07%/min; y‐intercept = 0.24; r^2^ = 0.19; *P* = 0.013), while no increase over time was seen for MOLLI 5s(3s)3s or SASHA (*P* = 0.127, and *P* = 0.216).

For volunteers, there was a smaller increase over time for MOLLI 5(3b)3 (0.05%/min; y‐intercept = 0.26; r^2^ = 0.09; *P* = 0.047) and MOLLI 5s(3s)3s (0.05%/min; y‐intercept = 0.25; r^2^ = 0.13; *P* = 0.015) but not for MOLLI 5(3s)3 or SASHA (*P* = 0.358 and *P* = 0.113, respectively). When excluding data from the first 10 min, there was still an increase in ECV over time for MOLLI 5s(3s)3s (0.07%/min; y‐intercept = 0.24; r^2^ = 0.13; *P* = 0.032) but not for MOLLI 5(3b)3, MOLLI 5(3s)3, or SASHA (*P* = 0.292, *P* = 0.163, and *P* = 0.106). No significant change or trend toward an increase or decrease in ECV fraction by radioisotope was seen over the first hour after termination (y = 0.17x; *P* = 0.728; *n* = 1).

### 3.5 Hct and heart rate

For results and discussion regarding Hct and heart rate, please see the Supporting Information, including Supporting Information Figures S1–S5.

## 4 DISCUSSION

The ECV of healthy myocardium, as measured by using pre‐ and postcontrast clinical T1‐mapping techniques, is consistent with results using a radioisotope‐based reference standard. Three variations of the MOLLI sequence showed a small overestimation of ECV, both when compared with SASHA and the reference method. SASHA shows no bias compared with the reference method. Additionally, ECV values of healthy myocardium were not found to be different between humans and pigs regardless of using MOLLI or SASHA.

Two studies published almost 2 decades ago by Arheden et al, using a similar radioisotope‐based reference method, found ECV of healthy myocardium in rats to be 16 ± 1% and 18 ± 1% by radioisotope and 23 ± 2% and 18 ± 1% (mean ± standard error of the mean), respectively, by CMR.^4^, ^21^ These studies, using long acquisition times precluding clinical use, measured T1 using an inversion‐recovery echo‐planar imaging sequence with gradient spoiling and repetition times of ≥7000 ms. Due to the long repetition time used, inversion‐recovery echo‐planar imaging does not require the Look‐Locker correction, similar to the SASHA sequence. It could, therefore, be that the current study is congruent with results of the second study from Arheden et al showing no statistically significant difference between CMR measurements and radioisotope.^21^ The reason for the difference in ECV seen between the 2 studies by Arheden et al is, however, not known. This strengthens the importance of verification data from large animal studies, which enables larger tissue volume sampling as well as more closely reflecting human physiology.

Flett et al used histological measurements of collagen fraction to validate the use of an early version of current clinically used T1 sequences.^8^ The correlation found between ECV by CMR and collagen fraction, later corroborated using ShMOLLI,^24^ was an important signal that ECV measurements by CMR can be used to detect differences in ECV. These results are further built on by the current study, which investigates the accuracy of ECV and seeks to verify the values.

The difference in native T1‐times seen between MOLLI and SASHA corroborates the results from several phantom‐studies showing that MOLLI underestimates T1 slightly while SASHA is more accurate.^10^, ^25^ Perhaps more importantly, the postcontrast myocardial T1 values were consistently lower using MOLLI than by using SASHA for both pigs and volunteers. This underestimation of T1 is likely the driver of the overestimated ECV in the current study and the higher ECV for MOLLI compared with SASHA also in previous work by Roujol et al.^10^

It should be noted that SASHA is also subject to effects causing underestimation of T1, albeit to a lesser degree than MOLLI, and that the 3‐parameter fitting for SASHA is the least sensitive to magnetization transfer.^15^, ^26^ To cause an increase in ECV, the effect of underestimating myocardial and blood T1 needs to result in a larger ratio between myocardium and blood (Equation 1). Indeed, some mechanisms shown to cause underestimation of T1 affect blood less due to longer T2‐times of blood compared with myocardium, which is in line with the results of postcontrast blood T1 values where no difference was seen between sequences. These mechanisms are a T2‐dependent effect on inversion efficiency, an effect of the balanced‐SSFP readout on the recovery curve, and magnetization transfer. In addition, the inflow of blood unaffected by any previous readout abolishes the need for $T_{1}^{*}$‐correction.^15^, ^27^

Thus, as per the section above, the higher ECV shown by MOLLI compared with SASHA is driven by lower myocardial postcontrast T1 values, which is incompletely compensated by lower precontrast myocardial T1 values. While precontrast blood T1 was lower for MOLLI compared with SASHA, it would not have a large enough role to explain any significant part of the difference as seen by the following example. Using Equation 2 and assuming a Hct of 31% and the following T1 values: T1_myocardial postcontrast_ 500 ms, T1_myocardial precontrast_ 1000 ms, T1_Blood postcontrast_ 300 ms, one finds that the difference between using a T1_Blood precontrast_ of 1600 ms (MOLLI) and of using T1Blood precontrast of 1820 ms (SASHA) is a change in ECV from 25.5% for MOLLI to 24.8% for SASHA.

The current study has implications for development of T1‐mapping techniques for measurement of ECV as well as for interpreting the results of currently used techniques. It can be argued that accuracy is not essential for separating health from disease in individual patients, as long as there are reference values for the particular setup used. However, accurate measurements and an understanding of what affects them are prerequisites for method standardization and for enabling comparison of values between different clinical centers, users, and scanners. Specifically, there is a need to understand the overestimation of ECV seen for MOLLI but not in SASHA and whether it can be corrected for.

### 4.1 Radioisotope reference method

99mTc is a commonly used radioisotope in clinical nuclear medicine due to a practical half‐life of around 6 h and for emitting photons at an energy level that falls within optimal range for gamma counters.^28^ As direct measurements of radioactivity rests on basal physical principles and can be measured with precision beyond the practical needs of macro‐physiology, it is hard to conceive of a theoretically more accurate reference method for our purposes. The criteria that need to be fulfilled for this to be true are (1) that the radiolabeled compound (99mTc‐DTPA) need to distribute in the predicted manner (i.e., extracellularly) and (2) the activity recorded must reflect the amount of isotope and not be influenced by self‐absorption or the shape of the sample. It has been shown, both for 99mTc‐DTPA and Gd‐DTPA, that the distribution is extracellular, and this fact is used in, for example, viability imaging in CMR.^29^, ^30^, ^31^, ^32^ As for self‐absorption, the half‐value layer of tissue for 99mTc seems to be around 4 cm which, if approximating the tissue to a cube with a density of 1 g/cm3, would correspond to a biopsy weighing 64 g.^33^ The average biopsy size being 0.25 g, we estimate that the comparative analysis between tissue and plasma is not sensitive to self‐absorption, as long as there is a roughly similar amount of volume in the samples.

### 4.2 ECV over time after contrast injection

Using MOLLI, it is necessary to allow sufficient recovery of magnetization between acquisitions to achieve accurate T1; therefore, the length of the pause between inversions affects the measurements. Thus, heart rate affects the result of MOLLI sequences, where the pause is based on the number of beats but not as much when the number of seconds is used. It also follows that longer T1 needs longer pauses to allow magnetization recovery, which is the basis for using sequences with longer pause precontrast than postcontrast. When time passes after contrast injection, T1 will gradually be prolonged, as Gd is eliminated by the kidneys and T1‐measurements could be affected depending on the length of the pause between inversions. An indication that this could in fact be the case is the increase seen in ECV over time for MOLLI 5(3b)3 and MOLLI 5(3s)3 for the pigs (Figure 3 of the main manuscript). These are the sequences with the shortest pause between inversions at the current heart rates (mean 88 ± 11 beats/min), and indeed it seems that the trend is for ECV by MOLLI 5(3b)3 to increase more over time after contrast than by MOLLI 5(3s)3 in animals. This effect was not as big in the volunteers as heart rates were closer to 60 beats/min meaning that the pauses and acquisitions defined by seconds were approximately as long as those defined by number of beats. Apart from the length of the pause between inversions, MOLLI is also sensitive to T2‐relaxation effects during the SSFP readout.^25^ As T2‐times are decreased due to the effect of Gd‐based contrast agent, there may be an underestimation in postcontrast T1 and, thus, a relative underestimation of myocardial ECV. Because the concentration of contrast agent is highest immediately after contrast administration the underestimation may be most pronounced then, and decreases over time.

The mechanisms described above are only applicable to the MOLLI sequences. As SASHA uses saturation recovery and crusher gradients the measurements are theoretically not dependent on the length of the pause in between saturation pulses because transversal magnetization does not carry over to the subsequent iteration of the sequence. In 2 previous studies, the timing of imaging after contrast was studied up to 25 min after contrast administration using slightly different MOLLI protocols compared with the current study.^7^ In one of the studies, there may be a trend toward increasing ECV with longer times after contrast administration,^22^ but none of the studies showed any statistically significant effect of timing after contrast administration on ECV, which is consistent with the findings in the volunteers of the current study.

Last, the inherent assumption in Equation 2 that a fast water exchange exists between the intracellular and the extracellular compartments may not hold true early on after contrast agent injection. It has been shown that, for these early measurements, using the fast exchange model can result in significantly underestimating the ECV in rats (by 80%) and in patients (by 67%).^34^ In our work, while underestimation of ECV on the order of 10% was seen early on after contrast administration, this was less than previously reported; perhaps as a result of using longer pauses with MOLLI. In any case, imaging 15‐25 min after contrast agent injection (Figure 5) may help in obtaining ECV measurements with the assumption of a fast water exchange, i.e., after the transient is over.

### 4.3 Limitations

The ECV measurements were tested in the normal range; therefore, no information is available on how accurate MOLLI or SASHA are in a wider range of ECV values. However, the clinical utility of these sequences is probably greatest in the range of ECV close to normal because a few percentage units in this range can indicate the difference between health and disease. The radioactivity of myocardium used for the reference method is measured in ex‐vivo biopsies, which could potentially affect the results. To minimize the potential error, care was taken to avoid the effect of capillary action on the myocardium and an experiment was performed showing that time from termination to biopsy had no effect on the measured ECV.

Similarly, it is not known what effect termination, with KCl infusion and the subsequent ventricular fibrillation, may have on ECV. However, KCl is routinely used in experimental studies to arrest the heart in diastole, and we believe it is the method of termination that preserves the physiological conditions best. Stated variability and precision of T1 and ECV estimates from MOLLI and SASHA may not be directly applicable to T1‐maps from the same sequences due to the use of ROI‐averaging for T1‐measurements in this study. However, reference values of precision for the 2 sequences were not part of the aims of this study and have been examined elsewhere.^10^

## 5 CONCLUSIONS

Clinically available MOLLI and SASHA techniques can be used to accurately estimate ECV in normal myocardium where MOLLI‐sequences show minor overestimation driven by underestimation of postcontrast T1 when compared with SASHA. The timing of imaging after contrast administration affected the measurement of ECV using some variants of the MOLLI sequence, which should be taken into account when planning clinical or research CMR protocols.

## CONFLICTS OF INTEREST

H.A. is stockholder in Imacor AB, Lund, Sweden. M.C. and H.E. have received consultancy fees from Imacor AB for analysis of cardiac MRI. No other authors report conflicts of interest.

## Supporting information


**FIGURE S1** R1 values in blood (left column) and myocardium (right column) before administration of contrast agent and over time after administration. Note the smaller spread in R1 after contrast in the pigs, which could be expected in the controlled experimental setting in young animals of the same weight and age. Note also that there are no obvious consistent differences in slope of the curves between volunteers and pigs. This indicates that there is no major difference in clearance rate of contrast agent between the groups
**FIGURE S2** Relationship between extracellular volume fraction (ECV) and heart rate for the different T1‐mapping techniques in human volunteers using data from different time points after contrast, thus the precontrast T1 measurement remains the same. The heart rate in one of the volunteers stood out as being higher than the rest, the data from this volunteer has been marked with a red circle. Results from all volunteers can be seen in the plots. Excluding the outlier resulted in the following nonsignificant slopes MOLLI 5(3b)3: y = 0.00026x+0.26, *P* = 0.746; MOLLI 5s(3s)3s: y = −0.0012x+0.34, *P* = 0.141; MOLLI 5(3s)3: y = 0.00020x+0.26; SASHA: y = 0.00056x+0.20, *P* = 0.479
**FIGURE S3** Relationships between extracellular volume fraction (ECV) and heart rate for the different T1‐mapping techniques in pigs using data from different time points after contrast, thus the precontrast T1 measurement remains the same. There was no statistically significant correlation between ECV and heart rate for any of the T1 mapping techniques
**FIGURE S4** Relationships between native T1 and heart rate for the different T1‐mapping techniques in pigs
**FIGURE S5** Relationships between native T1 and heart rate for the different T1‐mapping techniques in volunteers
**FIGURE S6** ECV as measured using postcontrast T1 from different time‐points after administration of contrast showing individual datapoints
**FIGURE S7** Postcontrast T1 values from porcine experiments for myocardium (2 left‐most columns) and for blood (two right‐most columns) showing data for each experiment individually. Note the consistently lower values of MOLLI sequences compared with SASHA for myocardium
**FIGURE S8** Postcontrast T1 values in volunteers for myocardium (2 left‐most columns) and for blood (2 right‐most columns) showing the data for each volunteer individually. Note the similar pattern as in Figure 4 where MOLLI sequences show consistently lower values than SASHA in myocardium

## References

[1] Kruhøffer P . Determination of Inulin in Urine and Plasma. Acta Physiol Scand. 1946;11:1–15.

[2] Bridge JH , Bersohn MM , Gonzalez F , Bassingthwaighte JB . Synthesis and use of radio cobaltic EDTA as an extracellular marker in rabbit heart. Am J Physiol. 1982;242:H671–H676. 6802001 10.1152/ajpheart.1982.242.4.H671 PMC3010220

[3] Tong CY , Prato FS , Wisenberg G , et al. Measurement of the extraction efficiency and distribution volume for Gd‐DTPA in normal and diseased canine myocardium. Magn Reson Med. 1993;30:337–346. 8412605 10.1002/mrm.1910300310

[4] Arheden H , Saeed M , Higgins CB , et al. Measurement of the distribution volume of gadopentetate dimeglumine at echo‐planar MR imaging to quantify myocardial infarction: comparison with 99mTc‐DTPA autoradiography in rats. Radiology. 1999;211:698–708. 10352594 10.1148/radiology.211.3.r99jn41698

[5] Arheden H , Saeed M , Higgins CB , et al. Reperfused rat myocardium subjected to various durations of ischemia: estimation of the distribution volume of contrast material with echo‐planar MR imaging. Radiology. 2000;215:520–528. 10796935 10.1148/radiology.215.2.r00ma38520

[6] Messroghli DR , Radjenovic A , Kozerke S , Higgins DM , Sivananthan MU , Ridgway JP . Modified Look‐Locker inversion recovery (MOLLI) for high‐resolution T1 mapping of the heart. Magn Reson Med. 2004;52:141–146. 15236377 10.1002/mrm.20110

[7] Lee JJ , Liu S , Nacif MS , et al. Myocardial T1 and extracellular volume fraction mapping at 3 tesla. J Cardiovasc Magn Reson. 2011;13:75. 22123333 10.1186/1532-429X-13-75 PMC3269374

[8] Flett AS , Hayward MP , Ashworth MT , et al. Equilibrium contrast cardiovascular magnetic resonance for the measurement of diffuse myocardial fibrosis: preliminary validation in humans. Circulation. 2010;122:138–144. 20585010 10.1161/CIRCULATIONAHA.109.930636

[9] Chow K , Flewitt JA , Green JD , Pagano JJ , Friedrich MG , Thompson RB . Saturation recovery single‐shot acquisition (SASHA) for myocardial T 1 mapping. Magn Reson Med. 2014;71:2082–2095. 23881866 10.1002/mrm.24878

[10] Roujol S , Weingärtner S , Foppa M , et al. Accuracy, precision, and reproducibility of four T1 mapping sequences: a head‐to‐head comparison of MOLLI, ShMOLLI, SASHA, and SAPPHIRE. Radiology. 2014;272:683–689. 24702727 10.1148/radiol.14140296 PMC4263641

[11] Engblom H , Kanski M , Kopic S , et al. Importance of standardizing timing of hematocrit measurement when using cardiovascular magnetic resonance to calculate myocardial extracellular volume (ECV) based on pre‐ and post‐contrast T1 mapping. J Cardiovasc Magn Reson. 2018;20:46. 29950178 10.1186/s12968-018-0464-9 PMC6022290

[12] Cerqueira MD , Weissman NJ , Dilsizian V , et al. Standardized myocardial segmentation and nomenclature for tomographic imaging of the heart: a statement for healthcare professionals from the cardiac imaging committee of the council on clinical cardiology of the American Heart Association. Circulation. 2002;105:539–542. 11815441 10.1161/hc0402.102975

[13] Kellman P , Wilson JR , Xue H , Ugander M , Arai AE . Extracellular volume fraction mapping in the myocardium, part 1: evaluation of an automated method. J Cardiovasc Magn Reson. 2012;14:63. 22963517 10.1186/1532-429X-14-63 PMC3441905

[14] Kellman P , Arai AE , Xue H . T1 and extracellular volume mapping in the heart: estimation of error maps and the influence of noise on precision. J Cardiovasc Magn Reson. 2013;15:56. 23800276 10.1186/1532-429X-15-56 PMC3702513

[15] Kellman P , Hansen MS . T1‐mapping in the heart: accuracy and precision. J Cardiovasc Magn Reson. 2014;16:2. 24387626 10.1186/1532-429X-16-2 PMC3927683

[16] Sörensson P , Heiberg E , Saleh N , et al. Assessment of myocardium at risk with contrast enhanced steady‐state free precession cine cardiovascular magnetic resonance compared to single‐photon emission computed tomography. J Cardiovasc Magn Reson. 2010;12:25. 20433716 10.1186/1532-429X-12-25 PMC2885384

[17] Nordlund D , Kanski M , Jablonowski R , et al. Experimental validation of contrast‐enhanced SSFP cine CMR for quantification of myocardium at risk in acute myocardial infarction. J Cardiovasc Magn Reson. 2017;19:12. 28132648 10.1186/s12968-017-0325-y PMC5278574

[18] Heiberg E , Sjögren J , Ugander M , Carlsson M , Engblom H , Arheden H . Design and validation of Segment–freely available software for cardiovascular image analysis. BMC Med Imaging. 2010;10:1. 20064248 10.1186/1471-2342-10-1 PMC2822815

[19] Xanthis CG , Nordlund D , Jablonowski R , Arheden H . Comparison of short axis and long axis acquisitions of T1 and extracellular volume mapping using MOLLI and SASHA in patients with myocardial infarction and healthy volunteers. BMC Med Imaging. 2019;19:18. 30795746 10.1186/s12880-019-0320-x PMC6387479

[20] Deichmann R , Haase A . Quantification of T1 Values by SNAPSHOT‐FLASH NMR imaging. J Magn Reson. 1992;96:608–612.

[21] Arheden H , Saeed M , Higgins CB , et al. Reperfused rat myocardium subjected to various durations of ischemia: estimation of the distribution volume of contrast material with echo‐planar MR imaging. Radiology. 2000;215:520–528. 10796935 10.1148/radiology.215.2.r00ma38520

[22] Ugander M , Oki AJ , Hsu L‐Y , et al. Extracellular volume imaging by magnetic resonance imaging provides insights into overt and sub‐clinical myocardial pathology. Eur Heart J. 2012;33:1268–1278. 22279111 10.1093/eurheartj/ehr481 PMC3350985

[23] Bland JM , Altman DG . Statistical methods for assessing agreement between two methods of clinical measurement. Lancet. 1986;1:307–310. 2868172

[24] Fontana M , White SK , Banypersad SM , et al. Comparison of T1 mapping techniques for ECV quantification. Histological validation and reproducibility of ShMOLLI versus multibreath‐hold T1 quantification equilibrium contrast CMR. J Cardiovasc Magn Reson. 2012;14:88. 23272651 10.1186/1532-429X-14-88 PMC3552758

[25] Gai ND , Stehning C , Nacif M , Bluemke DA . Modified Look‐Locker T 1 evaluation using Bloch simulations: human and phantom validation. Magn Reson Med. 2013;69:329–336. 22457268 10.1002/mrm.24251 PMC3826815

[26] Robson MD , Piechnik SK , Tunnicliffe EM , Neubauer S . T1 measurements in the human myocardium: the effects of magnetization transfer on the SASHA and MOLLI sequences. Magn Reson Med. 2013;70:664–670. 23857710 10.1002/mrm.24867

[27] Kellman P , Herzka DA , Hansen MS . Adiabatic inversion pulses for myocardial T1 mapping. Magn Reson Med. 2014;71:1428–1434. 23722695 10.1002/mrm.24793 PMC3775900

[28] Davey RJ , AuBuchon JP . Post‐transfusion red blood cell and platelet survival and kinetics: basic principles and practical aspects Blood Banking and Transfusion Medicine. Philadelphia: Elsevier; 2007:455–466.

[29] Aime S , Caravan P . Biodistribution of gadolinium‐based contrast agents, including gadolinium deposition. J Magn Reson Imaging. 2009;30:1259–1267. 19938038 10.1002/jmri.21969 PMC2822463

[30] Holness JL , Fleming JS , Malaroda AL , Warwick JM . 99mTc‐DTPA volume of distribution, half‐life and glomerular filtration rate in normal adults. Nucl Med Commun. 2013;34:1005–1014. 23880899 10.1097/MNM.0b013e328364aa12

[31] Gunasekera RD , Allison DJ , Peters AM . Glomerular filtration rate in relation to extracellular fluid volume: similarity between 99mTc‐DTPA and inulin. Eur J Nucl Med. 1996;23:49–54. 8586101 10.1007/BF01736989

[32] McAfee JG , Gagne G , Atkins HL , et al. Biological distribution and excretion of DTPA labeled with Tc‐99m and In‐111. J Nucl Med. 1979;20:1273–1278. 536795

[33] Fujimura N . The investigation of the absorption of 99mTc gamma‐rays by bone and soft tissue (author's transl). Radioisotopes. 1977;26:371–375. 578953 10.3769/radioisotopes.26.6_371

[34] Coelho‐Filho OR , Mongeon F‐P , Mitchell R , et al. Role of transcytolemmal water‐exchange in magnetic resonance measurements of diffuse myocardial fibrosis in hypertensive heart disease. Circ Cardiovasc Imaging. 2013;6:134–141. 23159497 10.1161/CIRCIMAGING.112.979815 PMC3587170

[35] Arai AE , Leung S , Kellman P . Controversies in cardiovascular MR imaging: reasons why imaging myocardial T2 has clinical and pathophysiologic value in acute myocardial infarction. Radiology. 2012;265:23–32. 22993218 10.1148/radiol.12112491 PMC3447177

