# Supplementary material for: Measuring extracellular volume fraction by MRI: First verification of values given by clinical sequences
Source: Magn Reson Med. 2019 Aug 16;83(2):662–72. doi: 10.1002/mrm.27938 (PMC6900009; doi:10.1002/mrm.27938)
Supplement: Supplementary file 1 — FIGURE S1 R1 values in blood (left column) and myocardium (right column) before administration of contrast agent and over time after administration. Note the smaller spread in R1 after contrast in the pigs, which could be expected in the controlled experimental setting in young animals of the same weight and age. Note also that there are no obvious consistent differences in slope of the curves between volunteers and pigs. This indicates that there is no major difference in clearance rate of contrast agent between the groups FIGURE S2 Relationship between extracellular volume fraction (ECV) and heart rate for the different T1‐mapping techniques in human volunteers using data from different time points after contrast, thus the precontrast T1 measurement remains the same. The heart rate in one of the volunteers stood out as being higher than the rest, the data from this volunteer has been marked with a red circle. Results from all volunteers can be seen in the plots. Excluding the outlier resulted in the following nonsignificant slopes MOLLI 5(3b)3: y = 0.00026x+0.26, P = 0.746; MOLLI 5s(3s)3s: y = −0.0012x+0.34, P = 0.141; MOLLI 5(3s)3: y = 0.00020x+0.26; SASHA: y = 0.00056x+0.20, P = 0.479 FIGURE S3 Relationships between extracellular volume fraction (ECV) and heart rate for the different T1‐mapping techniques in pigs using data from different time points after contrast, thus the precontrast T1 measurement remains the same. There was no statistically significant correlation between ECV and heart rate for any of the T1 mapping techniques FIGURE S4 Relationships between native T1 and heart rate for the different T1‐mapping techniques in pigs FIGURE S5 Relationships between native T1 and heart rate for the different T1‐mapping techniques in volunteers FIGURE S6 ECV as measured using postcontrast T1 from different time‐points after administration of contrast showing individual datapoints FIGURE S7 Postcontrast T1 values from porcine experiments for myocardium (2 left‐mos [file MRM-83-662-s001.docx]

**Supporting Information**

**Results**

*Hematocrit*

The mean hematocrit in pigs was 31±4%. There was no difference in hematocrit taken at baseline, 30 minutes after contrast injection (mean difference from baseline: 0.6%), or 60 minutes after contrast injection (mean difference from baseline: 0.8%) during the animal experiments (p=0.08). There was no difference in Hct as measured by Isotope or laboratory analysis (mean difference 0.6±1 percentage units, R=0.95).

*R1 and heart rate*

R1 values of time are presented in Figure S1. Results on the potential effect of heart rate on ECV are shown in Supporting Information Figure S2 and S3 while the effect on native T1 is shown in Supporting Information Figure S4 and S5.

**Discussion**

*Hematocrit*

As the formula for ECV includes 1-Hct, any changes in Hct may affect the accuracy of measurements and, if there are dynamic changes during imaging, accuracy may vary depending on when images are acquired. One reason for the serial sampling of Hct is the known effect of “spleen emptying” in response to stimuli such as breath-holding, hypoxia, and sympathetic activation wherein the red blood cells stored in the spleen are released into circulation, an effect that is especially pronounced in pigs, ^33–35^. As there was no change in Hct over time for the pigs this effect is not likely to have affected the results of the current study. The difference seen in Hct between pigs and human volunteers is congruent with what is previously known^36^.

**Supporting Information Figures**


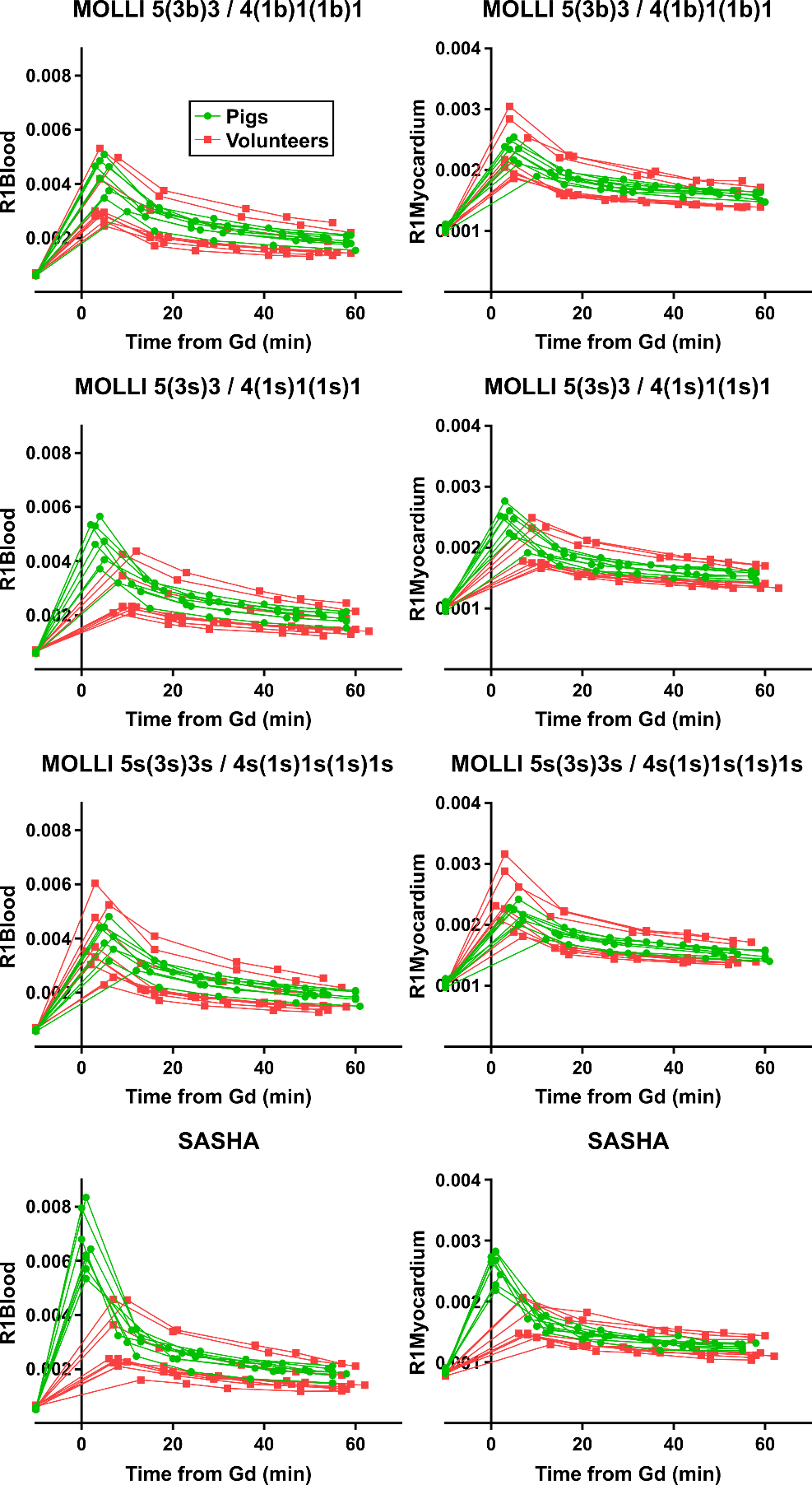


**Supporting Information Figure S1.** R1 values in blood (left column) and myocardium (right column) before administration of contrast agent and over time after administration. Note the smaller spread in R1 after contrast in the pigs, which could be expected in the controlled experimental setting in young animals of the same weight and age. Note also that there are no obvious consistent differences in slope of the curves between volunteers and pigs. This indicates that there is no major difference in clearance rate of contrast agent between the groups.


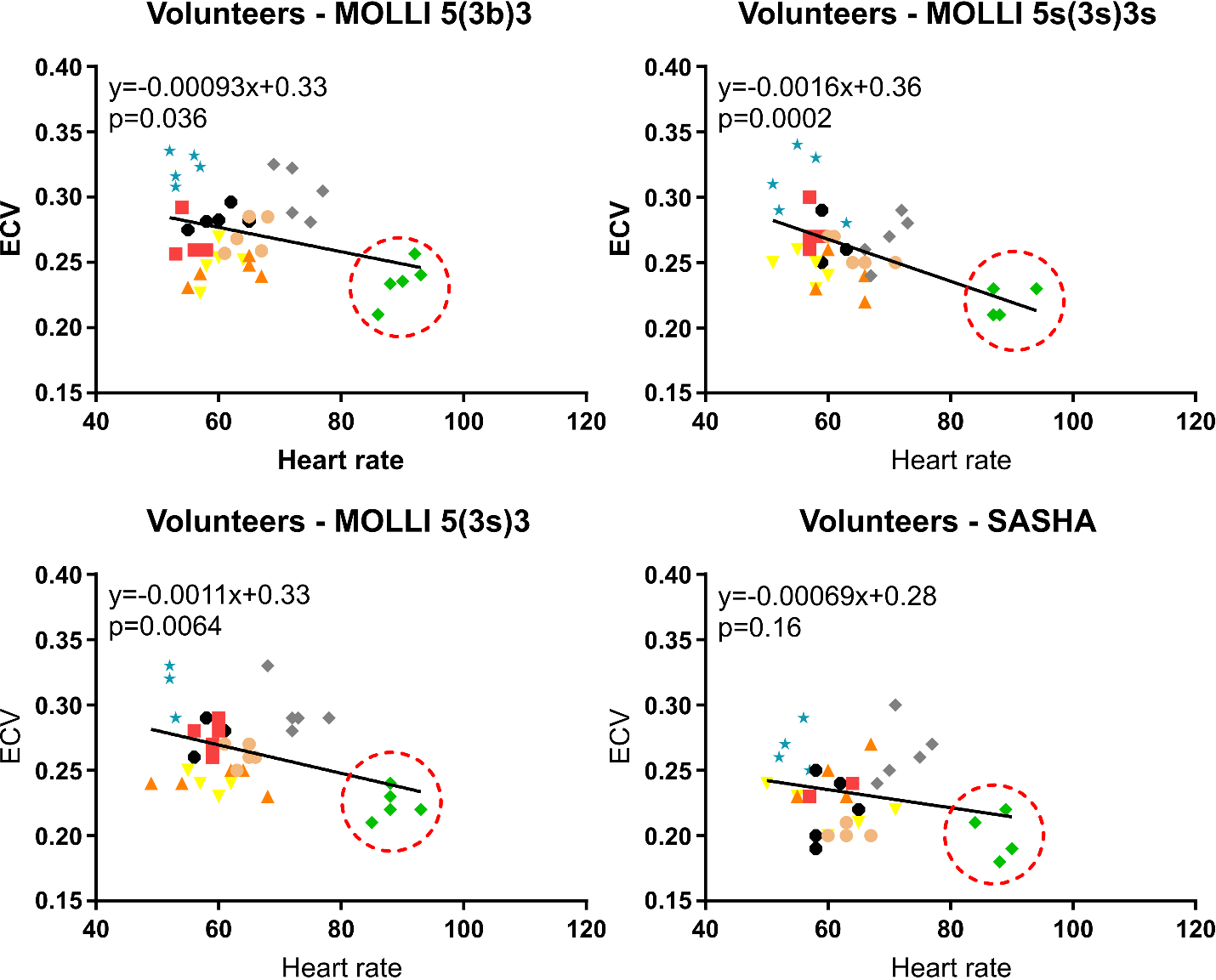


**Supporting Information Figure S2.** Relationship between extracellular volume fraction (ECV) and heart rate for the different T1-mapping techniques in human volunteers using data from different time points after contrast, thus the pre-contrast T1 measurement remains the same. The heart rate in one of the volunteers stood out as being higher than the rest, the data from this volunteer has been marked with a red circle. Results from all volunteers can be seen in the plots. Excluding the outlier resulted in the following non-significant slopes MOLLI 5(3b)3: y=0.00026x+0.26, p=0.746; MOLLI 5s(3s)3s: y=-0.0012x+0.34, p=0.141; MOLLI 5(3s)3: y=0.00020x+0.26; SASHA: y=0.00056x+0.20, p=0.479


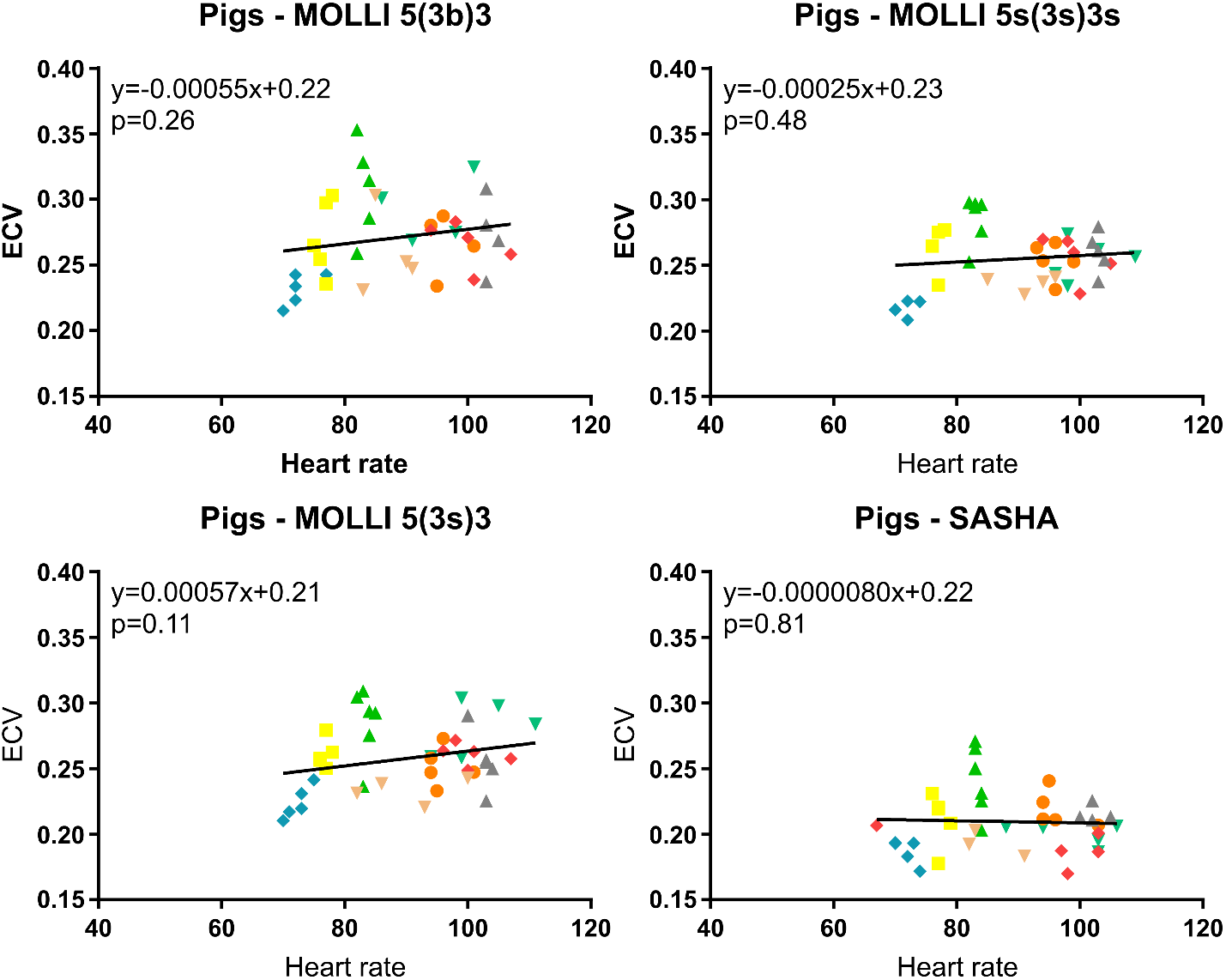


**Supporting Information Figure S3.** Relationships between extracellular volume fraction (ECV) and heart rate for the different T1-mapping techniques in pigs using data from different time points after contrast, thus the pre-contrast T1 measurement remains the same. There was no statistically significant correlation between ECV and heart rate for any of the T1 mapping techniques.


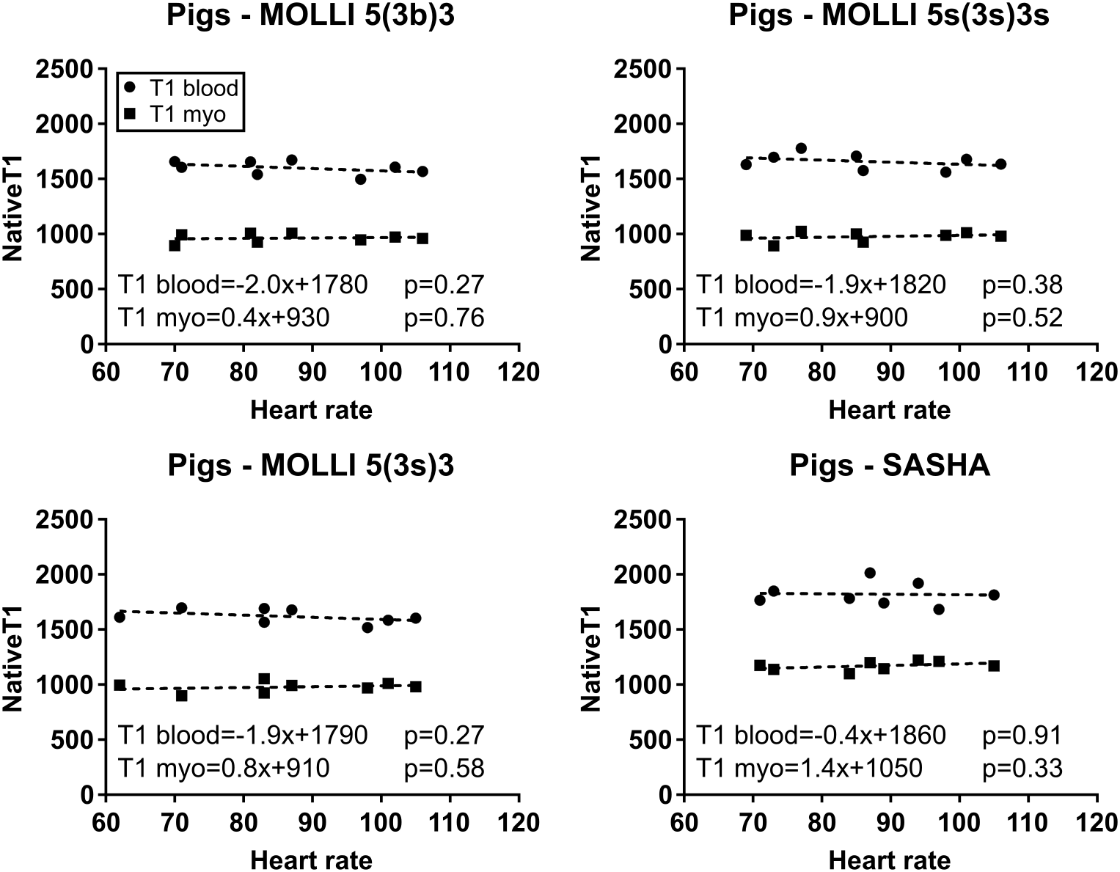


**Supporting Information Figure S4.** Relationships between native T1 and heart rate for the different T1-mapping techniques in pigs.


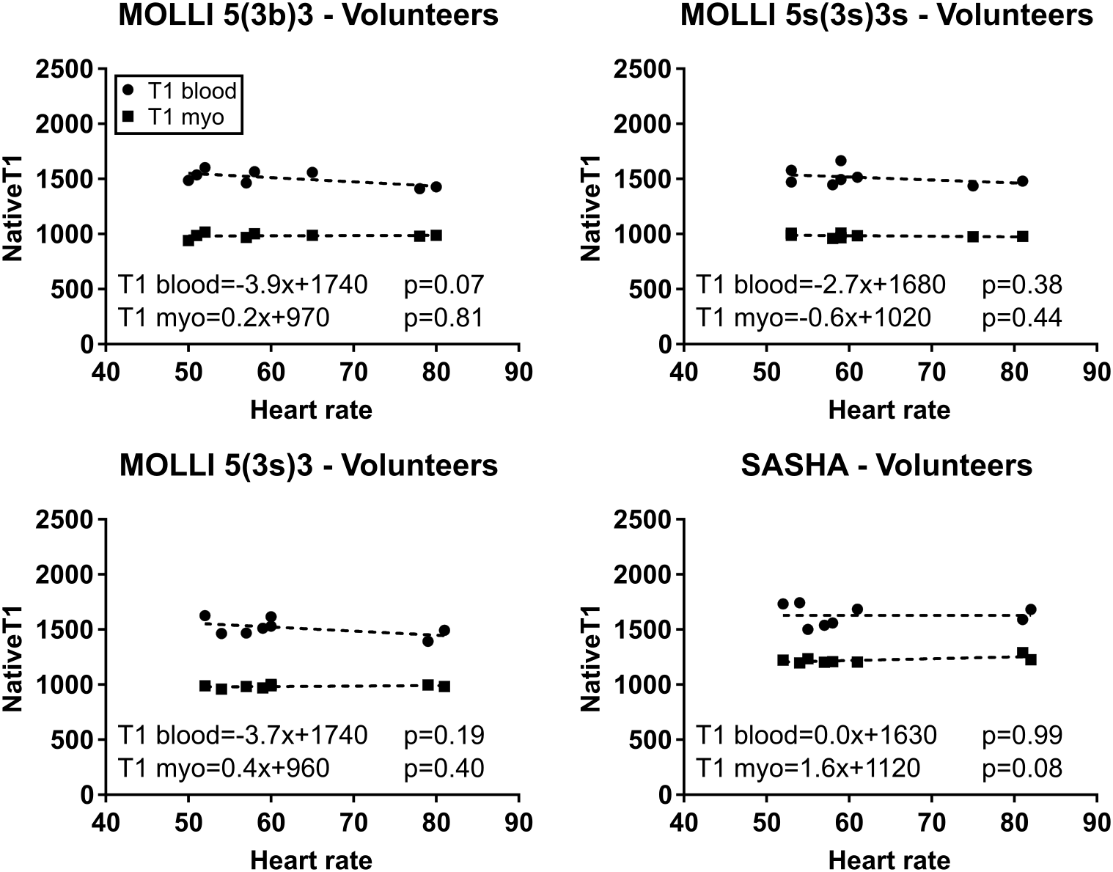


**Supporting Information Figure S5.** Relationships between native T1 and heart rate for the different T1-mapping techniques in volunteers.


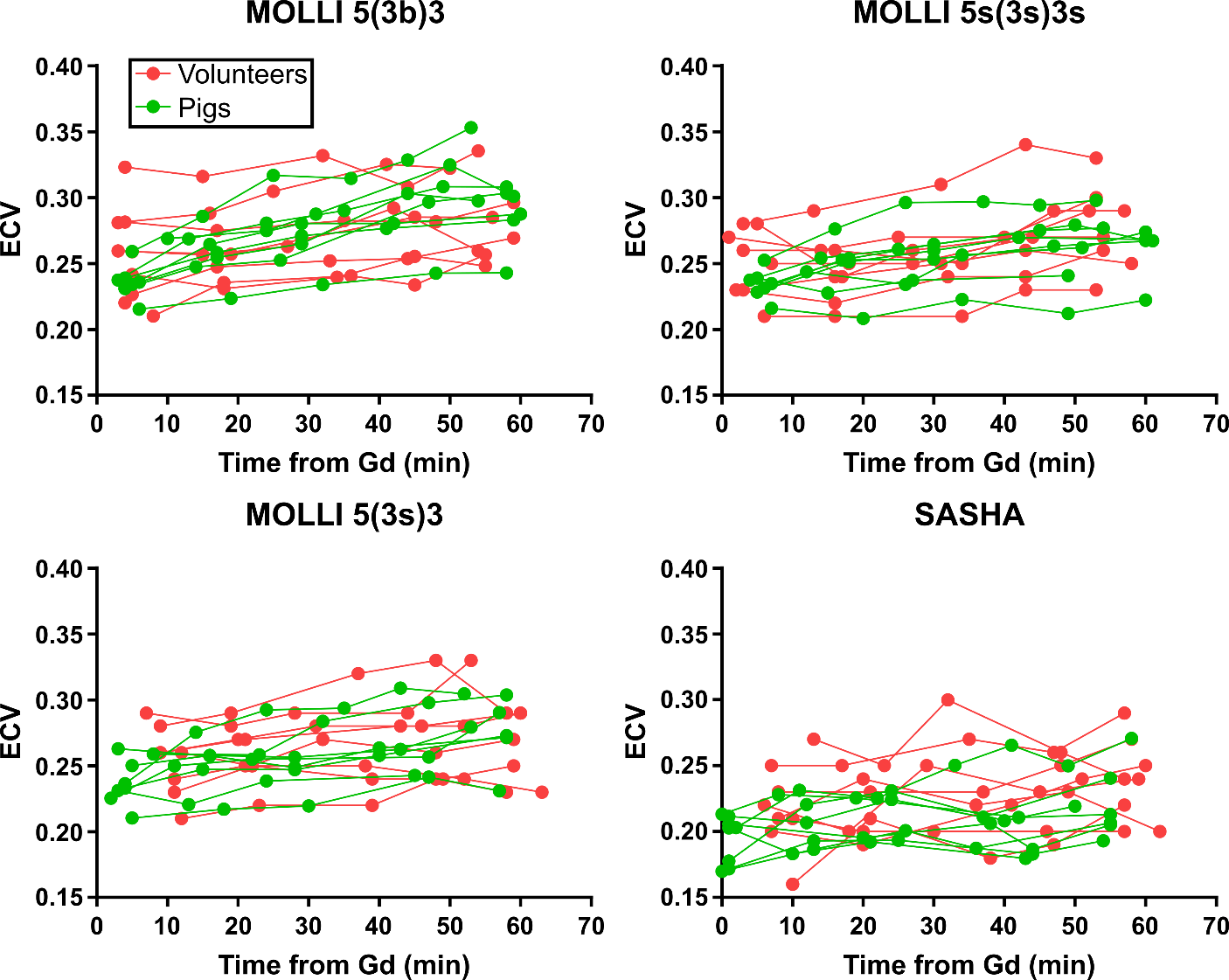


**Supporting Information Figure S6** ECV as measured using post-contrast T1 from different time-points after administration of contrast showing individual datapoints.


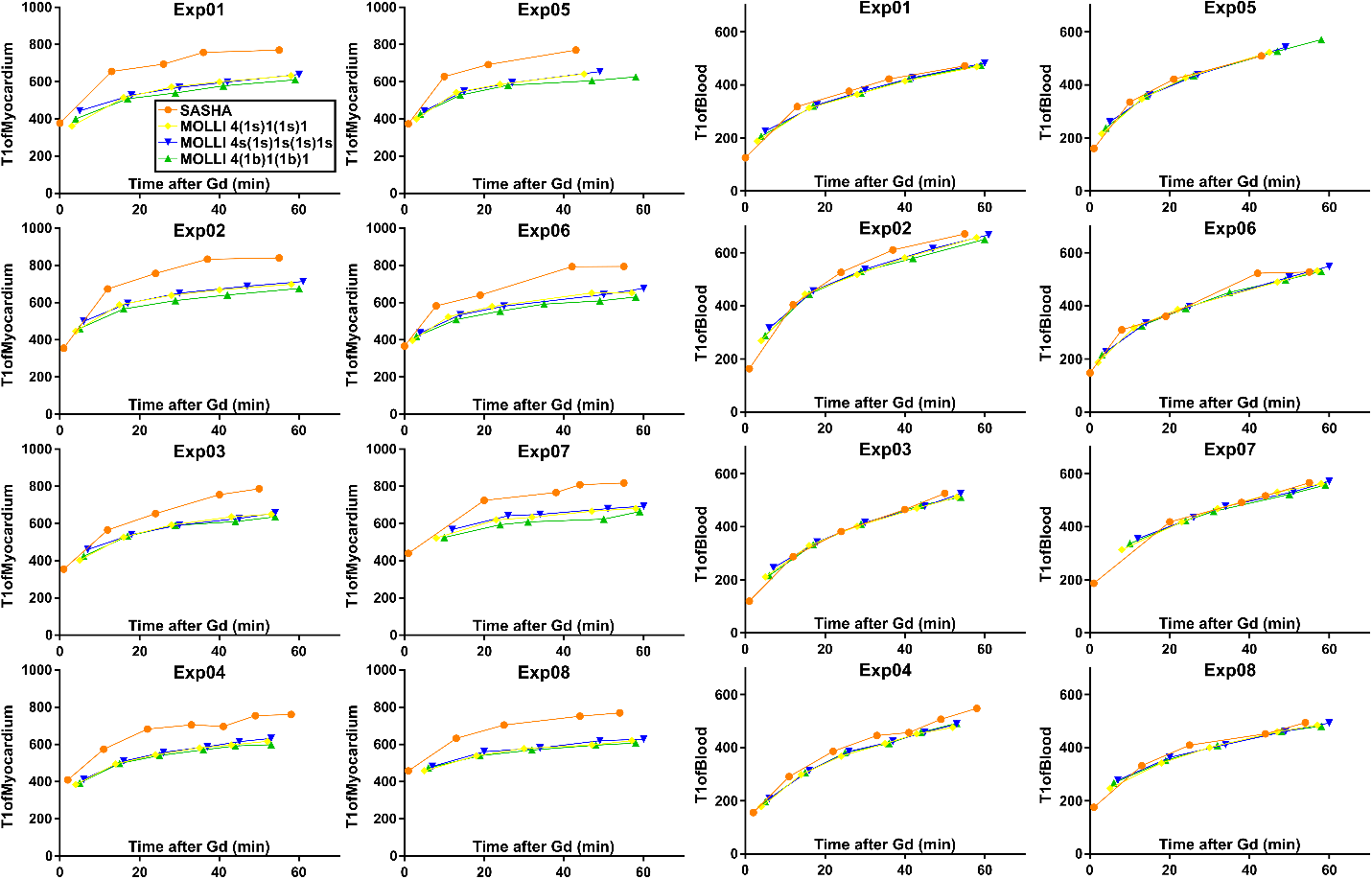


**Supporting Information Figure S7** Post-contrast T1 values from porcine experiments for myocardium (two left-most columns) and for blood (two right-most columns) showing data for each experiment individually. Note the consistently lower values of MOLLI sequences compared to SASHA for myocardium.


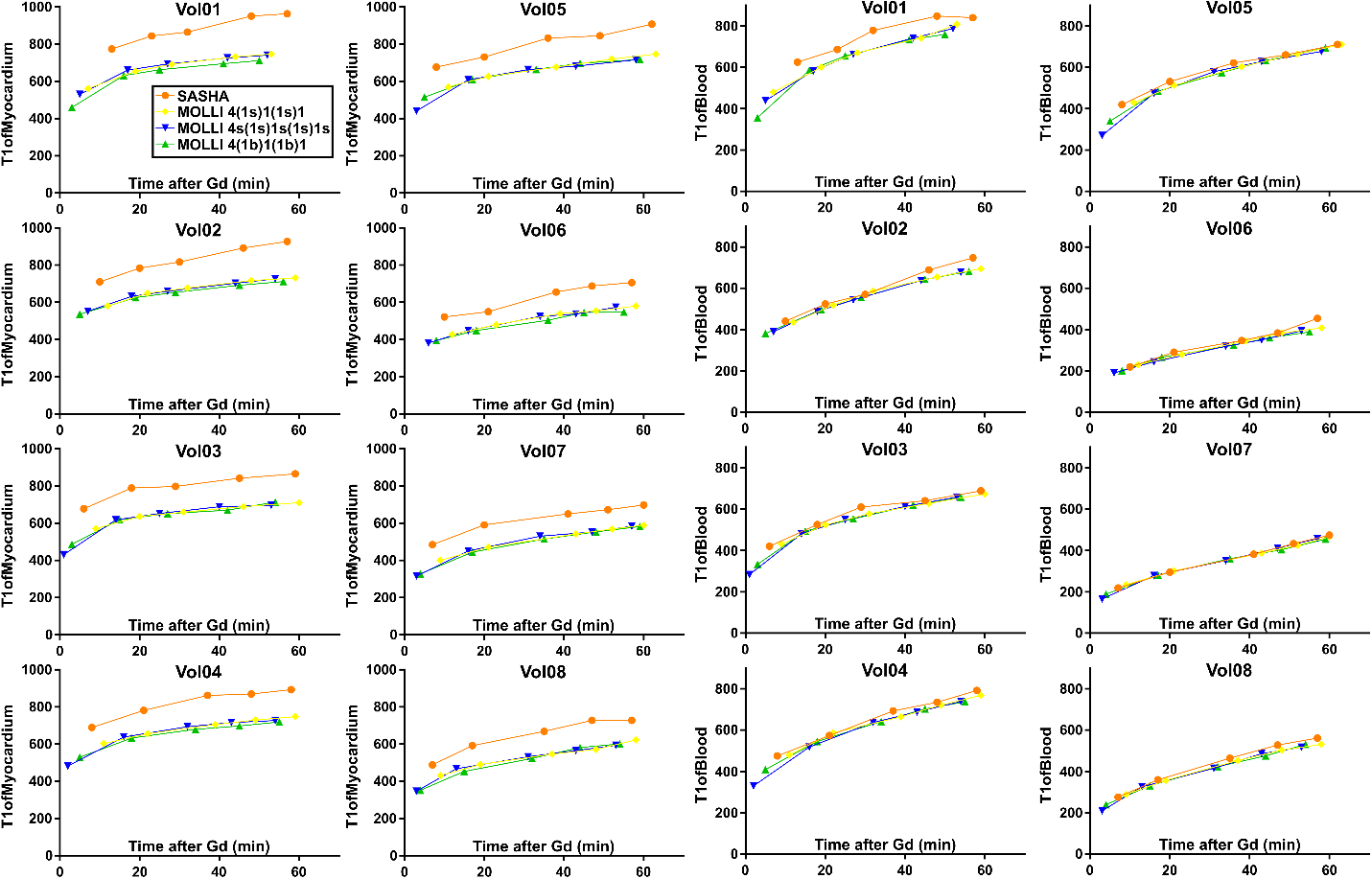


**Supporting Information Figure S8** Post-contrast T1 values in volunteers for myocardium (two left-most columns) and for blood (two right-most columns) showing the data for each volunteer individually. Note the similar pattern as in Figure 4 where MOLLI sequences show consistently lower values than SASHA in myocardium.
